# Opioid Regulation of Social Homeostasis: Connecting Loneliness to Addiction

**DOI:** 10.1016/j.biopsych.2024.11.011

**Published:** 2024-11-26

**Authors:** Guro Løseth, Marie Eikemo, Siri Leknes

**Affiliations:** Department of Psychology, University of Oslo, Oslo, Norway; Department of Psychology, University of Oslo, Oslo, Norway; Department of Physics and Computational Radiology, Oslo University Hospital, Oslo, Norway; Department of Psychology, University of Oslo, Oslo, Norway; Department of Physics and Computational Radiology, Oslo University Hospital, Oslo, Norway

## Abstract

Loneliness heightens the risk of substance use disorder, and a desire to escape this negative feeling motivates drug use. Opioid drugs in particular are believed to target neurobiological circuits involved in social bonding, increasing vulnerability to opioid addiction when social connectedness is lacking. In this narrative review, we consider how current understanding of μ opioid modulation of reward and threat processing across domains sheds light on the mechanisms that link loneliness and substance use. We discuss evidence for state- and context-dependent μ opioid modulation of social affect and behaviors, which appears to promote prioritization of high-value reward options also in the context of threat. Tying this literature to the model of social homeostasis, we argue for a role of μ opioids in regulating social homeostasis across species. Finally, we explore how disruption of social homeostasis in chronic opioid use contributes to continued drug use. We highlight how increasing patients’ psychosocial resources and opportunities for social bonding can improve recovery from drug addiction. Throughout, we consider the translational robustness and generalizability of the nonhuman animal evidence in light of existing human research.

In March 2020, governments around the world enforced strict social distancing to prevent spread of the COVID-19 corona virus. During the following months, a spike in drug-related deaths—mainly opioid related—was observed in many countries, including the United States and Norway (1–3). However, a similar overdose spike was not observed in Sweden (see Figure 1), a country with comparable wealth and public welfare similar to Norway’s, but where no lockdown was enforced. While several factors may have contributed to increases in drug-related deaths during the pandemic, including restricted access to treatment and changes in drug availability and purity, it seems possible that the severity of social restrictions was among the driving factors. Analogously, the dramatic rise in drug-related deaths of despair seen in the United States over the past few decades (4) has been attributed to living conditions that undermine social connectedness—including poor community support, extended working hours, and minimal paid leave—leading to increased loneliness, particularly in groups with lower socioeconomic status (5).

Social disconnection increases risk of substance use disorder, depression, and anxiety (6) as well as pain (7). Therefore, social restrictions imposed during the pandemic risked exacerbating 3 other ongoing global epidemics: those of loneliness (8), chronic pain (9), and opioid misuse (10). Cross-sectional studies reported increased prevalence of loneliness, pain, and substance use during the first years of the pandemic (11– 13). While the opioid crisis was initially sparked by over-prescription of opioids to treat pain (14), social disconnection added fuel to the fire because people tried to mitigate loneliness with substance use (15). Several studies have found that participants report using substances to relieve loneliness (16–18), but research on how loneliness impacts concurrent substance use is limited. An online diary study of substance use in 2687 U.S. adults during the COVID-19 pandemic reported that feeling more lonely was associated with increased alcohol and noncannabis drug intake on the same day (19). In patients with chronic pain, negative affect predicted opioid craving (20,21), and loneliness predicted daily opioid use (22).

The desire to escape negative feelings is a primary motivation for substance use (23). Loneliness is a highly aversive state that is thought to have evolved to promote affiliative behaviors (24), which are life supporting in social animals (25). Defining loneliness as an emotional marker of unmet social needs, the model of social homeostasis (see Figure 2) proposed by Tye and colleagues (26,27) posits that imbalances in social needs are regulated similarly to physiological imbalances, where regulatory systems strive to maintain a steady state that enables optimal functioning of the organism (28). In this narrative review, we explore the links between social behaviors, loneliness, and opioids, focusing on the main target of opioid drugs: the μ opioid receptor system. We discuss how the μ opioid system modulates reward and threat across various domains, potentially influencing social homeostatic processes. Then, we consider how social connectedness is challenged in opioid use disorder (OUD) and how substance use treatment may capitalize on social homeostatic mechanisms by integrating social interventions.

## Social Connection And Disconnection In Light Of Reward And Threat Processing

Strong connections to favored social partners increases fitness and survival across taxa (29–32). Humans have a fundamental need to belong (33). During infancy, we are completely reliant on social care to regulate our emotional and physiological state. Our brains develop within this context to expect a certain access to social support—a social baseline (34), which forms a predictive model of the world where we engage in reciprocal regulation of physiological and emotional states through social communication (35). Social isolation and loneliness—a perceived lack of social resources—thus represent deviations from the expected social baseline (34). The quality of social relationships appears to calibrate social reward and threat sensitivity (36,37) and to form a central part of the bioenergetic resources that people count on to cope with challenges (34). Emerging evidence linking social inclusion to lower basal glucose levels (38) and loneliness and social avoidance to higher levels of glucose and sugar intake (39,40) supports theories of socially regulated allostasis: prediction of energy demands (41,42). Social isolation increased fatigue more than food deprivation (43). The increased toll on individuals left to cope with life’s challenges on their own is reflected in their heightened risk of mortality and morbidity (44–46), including metabolic disorders (47).

Restoring high-quality social connection is the most sustainable strategy to remedy loneliness. However, when threat of rejection is perceived as high, people tend to distance themselves from others (48). Fear of social rejection is a potent driver of behavior (49), and if perceived opportunities for social support are lacking, substances that offer short-term relief may seem like an attractive escape.

### The Brain Opioid Theory Of Social Attachment

The abuse liability of opioid drugs has been theorized to result from their actions on neurobiological circuits that evolved to encode soothing social contact. According to the brain opioid theory of social attachment (50), formation and maintenance of social bonds is promoted by endogenous opioid signaling causing positive emotions in response to positive social interaction. On the other hand, it has been proposed that social deprivation reduces opioid tone and produces aversive withdrawal-like symptoms. Accordingly, opioid drugs could grant temporary relief and mask the need for social connection by binding to the receptors that underpin social motivation, potentially hijacking the system to promote drug seeking instead of social attachment (51).

Substantial evidence from rodents and nonhuman primates supports central tenets of the theory (52). For example, μ opioid signaling is necessary for establishment of social attachment (53,54) and socially conditioned place preference (55,56). Following distressing social isolation, behaviors aimed at soliciting soothing social contact and proximity to safe others are typically reduced by opioid agonist drugs and increased by antagonists (57). Antagonist drugs have also been shown to prevent the soothing effects of social reconnection (58). However, key premises of the theory such as the μ opioid dependence of soothing effects of social support and reconnection have yet to be tested in humans.

Nevertheless, much of the human literature is consistent with the idea that the μ opioid receptor system modulates social reward and threat, although effects are typically smaller and more varied than in the rodent literature. Most people seek social connection when the perceived benefits outweigh the risks of rejection. If fear of rejection prevails, social avoidance may be preferred even if it leads to loneliness. In the following, we explore the broader role of the μ opioid system in threat and reward processing, which, while not limited to the social domain, is key to understanding its impact on social homeostasis. After reviewing the nonhuman animal evidence, we consider the human studies and then outline how these processes may influence the mechanisms of social homeostasis.

### State- And Context-Dependent μ Opioid Modulation Of Reward Across Domains In Preclinical Models

The reward value of a cue or opportunity depends on how well it corresponds to the current motivational state and homeostatic needs of the organism (59,60). The overall function of the μ opioid system appears to be to direct the organism toward the most valuable option in any given context (61,62). The endogenous μ opioid system is widely reported to enhance reward responsiveness across social, food, drug, and other domains (63,64). For example, μ opioid signaling in the rodent striatum promotes motivated behaviors such as food intake (65), drug seeking (66), pair-bond formation (67), and social play (68). However, baseline behaviors in nondeprived animals such as consumption of normal foods (69) or engagement in social behaviors such as huddling (53) or parental care (55) do not rely on μ opioid signaling. Instead, μ opioid activity promotes the preferential increase in motivation for and hedonic responses to higher-reward options, such as 1) consumption of chocolate relative to normal food (70–72), 2) engagement in favored social activities such as play relative to nonplay in juveniles (73), 3) preferential mating with a partner (in socially monogamous voles) (67), or 4) with fertile individuals (in mice) (74). These and other rodent studies have shown μ opioid promotion of the most rewarding opportunity available (72).

Determination of reward value is also contingent on homeostatic needs, as exemplified by hunger-induced increases in food reward (60,75). Recently, hunger-enhanced eating was found to depend on a specific μ opioid microcircuit projecting from the dorsal raphe nucleus to the nucleus accumbens (76). The emerging pattern suggests that μ opioid promotion of approach and prioritization of higher-value rewards happen in a state-dependent manner. Specific circuits that underpin isolation-induced increase in motivation for social connection have yet to be described (77), but they likely involve dopamine neurons in the dorsal raphe nucleus that promote sociability and negative affect following acute isolation (78). Because social interactions can represent threat as well as reward, the weighing of reward value relative to threat is pivotal in social decision making. In rodents, there is evidence that the μ opioid receptor system plays a modulatory role in navigating these competing affective dimensions.

### μ OPIOID MODULATION OF THREAT AND REWARD EXPLORATION ACROSS DOMAINS IN PRECLINICAL MODELS

μ Opioid signaling modulates adaptive responses to threat across domains (63). For example, learning from fear-inducing and threat-related cues (fear conditioning) is impeded by μ opioid activation (79). Social exploration, which is promoted by endogenous opioids in rodents and primates (80–84), typically depends on a willingness to take risks. Therefore, μ opioid activity could promote exploration of potential rewards and identification of the most valuable reward options through enhanced reward sensitivity, decreased threat sensitivity, or both. We note that opioid activity boosts social interaction in contexts in which the animals would otherwise show reticence (85), consistent with μ opioid downregulation of threat sensitivity. Similarly, as described in Fields’ motivation-decision model of pain (86), endogenous opioid activity promoted prioritization of potential food reward (chocolate) in a risky setting (hot plate) (87).

We have previously compared opioid effects on social motivation in the contexts of nondistressing and distressing social isolation and revealed strongly state-dependent effects (88). A seemingly paradoxical pattern emerged where μ opioid effects on social approach shifted from promotion to inhibition when animals were socially deprived. This shift could reflect opioid regulation of threat, such that opioid drugs blunt the affective aspect of social deprivation. Unfortunately, relatively few human studies have addressed state-dependent opioid effects. Below, we review the evidence for acute opioid effects on threat and reward responses in healthy, nondeprived humans.

### Opioid Modulation Of Reward And Threat In Healthy Humans

In contrast to the array of advanced pharmacogenetic approaches and state manipulations that have been used to probe opioid-sensitive microcircuits in nonhuman species, human opioid research has primarily relied on systemic drug studies under nondeprived conditions. Note that whereas common analgesic medications primarily activate the μ opioid receptor, medications used to uncover the role of endogenous opioids have nonspecific opioid receptor binding. We discuss generalizability from nonhuman to human evidence in Box 1.

Overall, the human psychopharmacological evidence is consistent with the preclinical findings that opioid signaling promotes reward by increasing preference (expressed as subjective enjoyment or behavioral engagement) for high-reward stimuli relative to lower-value alternatives across the domains of social reward (89–92), taste reward (93,94), and monetary reward (95,96). These effects are typically small and variable, however. Several human studies have reported intact subjective liking and wanting of social and nonsocial rewards after full opioid blockade (94,97–100). Some null findings were nevertheless accompanied by changes in neural responses (100–102), reduced facial reactions (94,103), or suppressed pupil dilation (98), consistent with a possible subtle inhibition of reward processing after opioid blockade.

Opioid manipulations of threat sensitivity in humans have revealed patterns consistent with the rodent evidence, but this literature is also characterized by modest and variable effects. Opioid agonists caused a small decrease in anticipatory anxiety of public speaking (104,89) but did not improve negative affective responses to stress induction tasks in 4 studies of healthy humans (104–106,89). Nevertheless, behavioral and physiological measures of threat sensitivity have revealed patterns consistent with the rodent evidence. Notably, antagonist studies have shown increased sensitivity to nonsocial (abstract) threat cues (107,108). Two positron emission tomography studies have also linked sensitivity to social rejection to μ opioid signaling in healthy humans (109) and humans with depression (110). Studies of opioid regulation of reward behaviors in the context of threat in humans are scarce, but in direct contrast with the rodent findings, one study found intact analgesia from high-value social stimuli (erotic pictures) after opioid blockade (111). However, social exploration in the form of eye-gaze patterns to photographs was promoted by morphine and reduced by opioid blockade in a no-risk context (91), consistent with μ opioid promotion of social exploration in rodents (112). Further research using more naturalistic measures and varied contexts is needed to tease out the role of endogenous and exogenous opioids in regulating how people navigate their complex, rewarding, but sometimes risky social environments. In the following sections, we discuss potential roles of μ opioid activity in social homeostatic processing in humans.

### Potential Roles Of The Opioid System In Social Homeostasis

**Figure 2:**
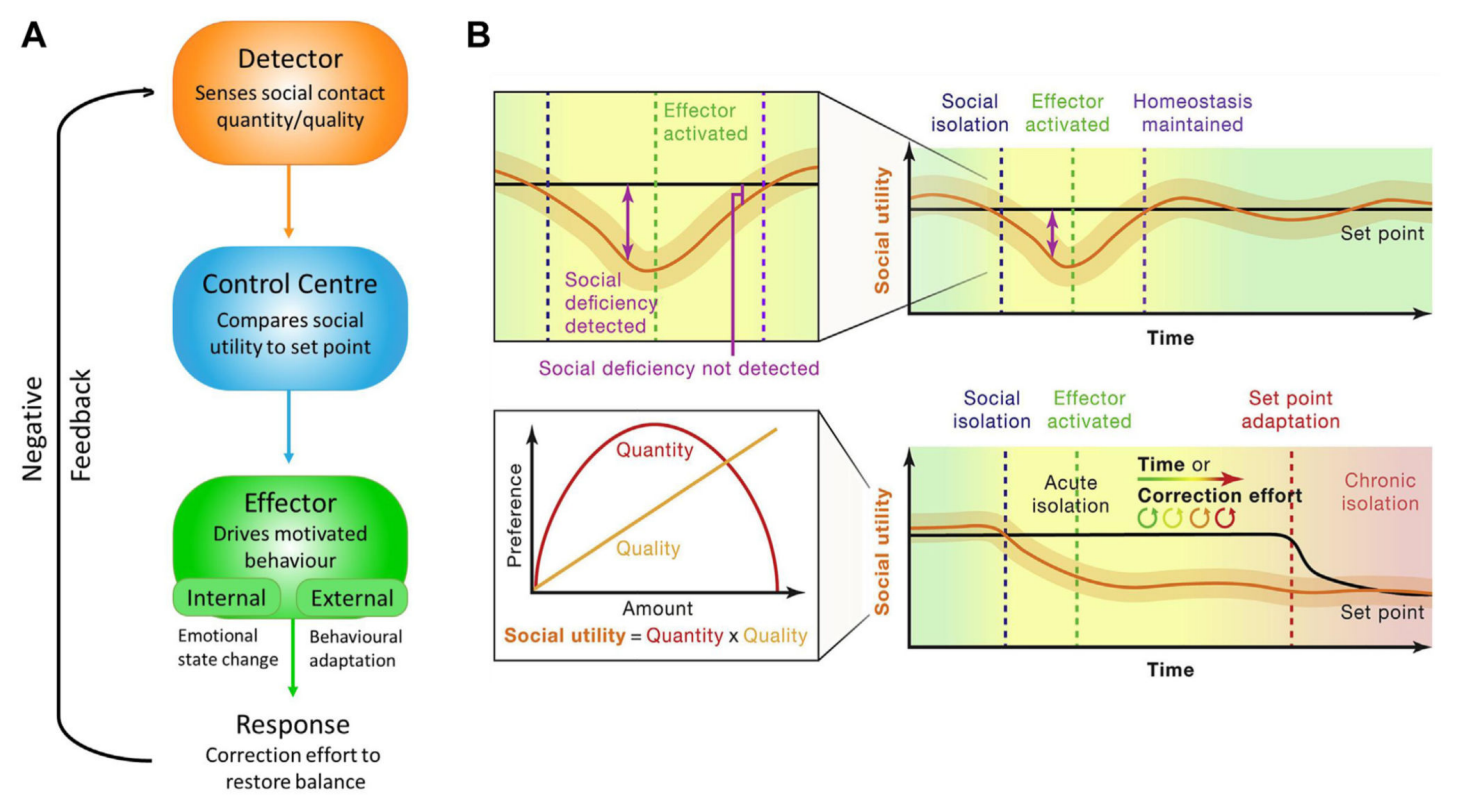
The model of social homeostasis. (A) The model of social homeostasis proposed by Tye and colleagues (26,27) suggests that social motivation is governed by a balancing system consisting of 3 main components—a detector, a control center, and an effector component. [Panel **(A)** is adapted with permission from Matthews and Tye (27).] **(B)** Changes in the quality and quantity of social contact are sensed by the detector and then evaluated by a control center that compares the social utility to the optimal set point before relevant effector systems are set in motion to counteract deviations from set point and reinstate equilibrium. When social homeostasis is disrupted, internal regulation could take the form of emotion regulation and could involve strategies such as cognitive reappraisal (162), e.g., “they are probably stressed, that’s why they were short with me,” or memory retrieval that invokes emotional proximity to internalized attachment figures (163). External effector systems involve behavioral adaptations to reduce the discomfort of loneliness, such as seeking social contact or other sources of relief (27). When deficiencies (or surplus) in social contact are long lasting and correction efforts are unsuccessful, set point could also be adjusted to better correspond to the social environment (26). The dynamic nature of the set point in this model suggests that social homeostasis could also be considered within a settling-point or allostatic framework (42,164). [Panel **(B)** is adapted with permission from Lee *et al*. (26).]

In this section, we discuss how opioid mechanisms may influence processes related to the 3 main components of social homeostasis (see Figure 2 for a description).

#### Detector

When encountering others, the detector component takes in a multitude of cues that pertain to their identity, gender, age, size, fitness, and social status. These cues help determine their reward value, e.g., how desirable they are as a sexual partner or social ally or how well they would satisfy other (social) needs. The same cues also inform predictions about the like-lihood of rejection or aggression if approached. We propose that the affective dimensions of reward and threat appraisal form a central part of detecting the quality of a social interaction, and thus for determining social utility. These affective dimensions are likely modulated by the opioid system, consistent with its described role in state-dependent and context-sensitive modulation of reward and threat. Preliminary evidence in humans is consistent with a role for the opioid system in enhancing sensitivity to positive social cues and decreasing social threat perception. For example, small doses of μ opioid agonists have been found to preferentially enhance attractiveness ratings for the most attractive faces (92) and reduce perception of subtle emotional cues that signal threat such as anger (113) and fear (89,114). However, this literature is small, studies lack statistical power, and replication is needed.

We also note that detection of new social opportunities such as a sudden access to potential sexual partners should elicit a social need (social desire) without concurrent homeostatic deviations. Social exploration, promoted by μ opioid signaling, would enable both identification (detector mechanisms) and approach (through effector mechanisms) of such opportunities.

#### Control Center

The proposed control center determines how well the social utility of the current context answers to the social needs and homeostatic set point of the individual. If it matches well, the individual is in social homeostatic balance. In a recent review, Bales *et al*. suggested a role for factors such as sociability, attachment style, and genetics in shaping individual set-point differences (115). Several lines of evidence tentatively link the μ opioid system to such factors.

μ Opioid receptor knockout mice (*Oprm1*−*/*−) display deficient social reward processing, expressed for example as lowered social interest and impaired socially conditioned preference formation and attachment (54,55,116). This suggests that μ opioid signaling may be necessary for a higher social homeostatic set point. However, acute pharmacological suppression of opioid signaling in nondeprived individuals does not appear to perturb social homeostatic balance to any great degree. A series of opioid antagonist studies only found small although relatively consistent reductions in self-reported social connectedness in nondeprived people (117). For example, viewing pictures of close others increased feelings of social connectedness and activity in the ventral striatum (118), both of which were reduced but not eliminated by opioid blockade (119). Cross-sectional studies have pointed to disruptions in the μ opioid system in people with chronic pain (120,121) and poor mental health (122–128). The long-term consequences of disrupted μ opioid functioning for social connectedness remain an open question. It is a possibility that subtle acute effects accumulate over time to cause anhedonia and a lowering of social homeostatic set point.

Homeostatic set point could also be underpinned by attachment style. Differences in social attachment patterns across vole species and human individuals have been linked to μ opioid receptor density (129–131). Furthermore, some candidate gene studies have linked a functional polymorphism of the μ opioid receptor gene (*OPRM1* A118G) to human social anhedonia (132), avoidant attachment, and responsiveness in close relationships (133,134). However, these findings have not been replicated, and the evidence is insufficient to draw conclusions. A study conducted with a substantially larger sample did not find associations between the A118G polymorphism and variations in social rejection sensitivity (135).

#### Effector

The effector component evokes behavior and internal regulation to minimize deviations from homeostatic balance. To avoid departures from optimal conditions, effector mechanisms should come into effect at an early stage—before negative affect dominates the motivational state. Isolation-induced increase in social play and exploration in non-distressed juvenile rats is an illustration of early effector activation (84). As reviewed above, this response is likely promoted by μ opioid enhancement of appetitive social motivation via upregulation of reward responsiveness and downregulation of threat appraisal, impacting internal effector mechanisms such as emotion regulation as well as external effector mechanisms that initiate social behavior.

In contrast, following distressing isolation, social exploration is suppressed, and social approach is directed toward safe and familiar others. This aversive social motivation is typically intensified by opioid blockade and blunted by opioid drugs (88), but the precise mechanisms involved have yet to be revealed. We hypothesize that opioid receptor activity modulates the dorsal raphe nucleus dopamine mechanism described to underpin social approach driven by aversive motivation (78). One possibility could be that this circuit is inhibited by tonic opioid signaling that is lifted in response to a social deficit or distress state. In this case, blocking relevant opioid receptors should lift this brake artificially. Conversely, agonist stimulation should have the potential to keep the brake on even during severe deficits or distress.

**Figure 3:**
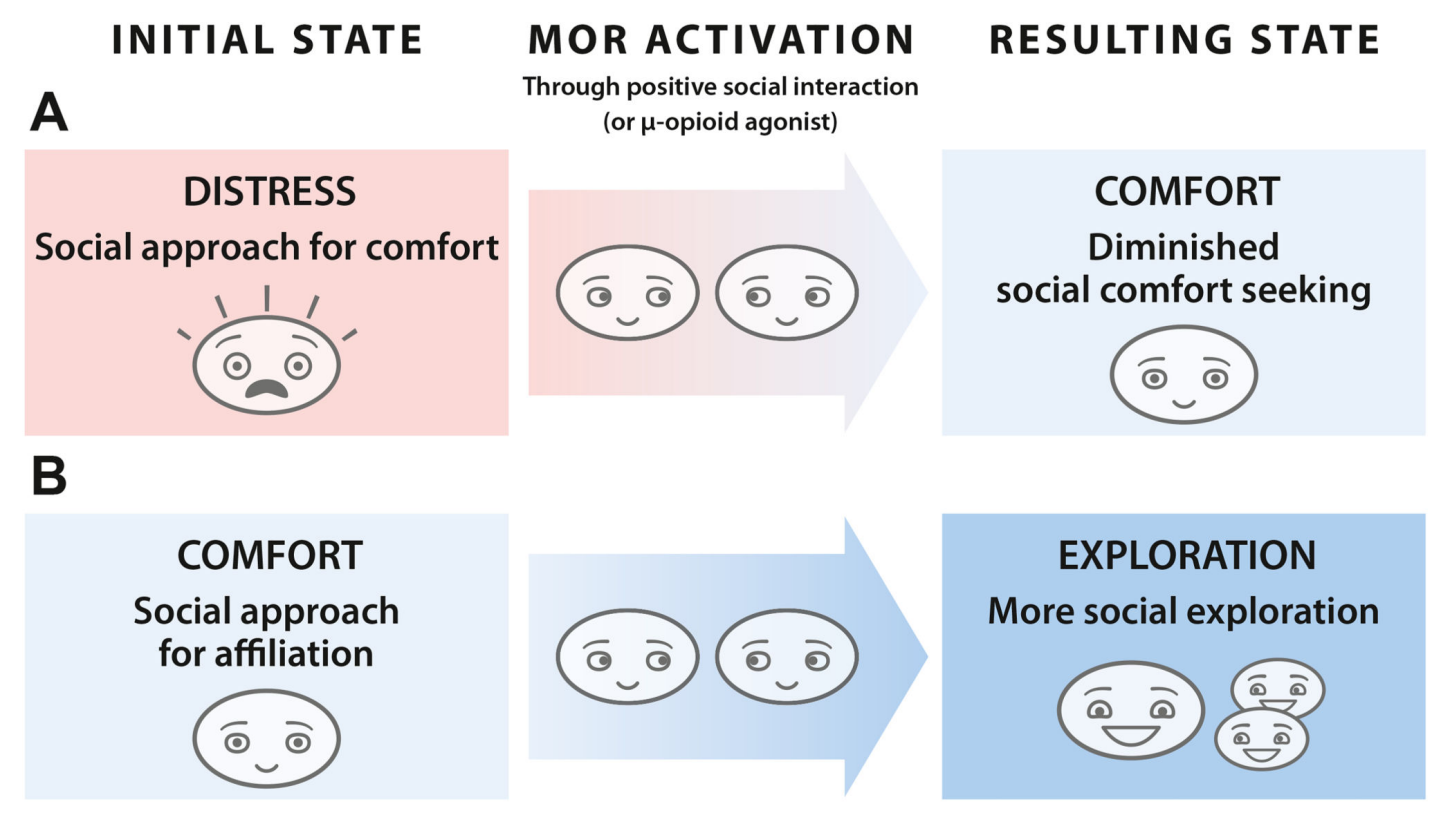
The state-dependent μ opioid modulation of social motivation (SOMSOM) model. **(A)** In a state of separation distress, aversive motivation drives individuals to seek social contact for comfort and relief. According to SOMSOM, this process is modulated by μ opioid receptor (MOR) signaling. This postulation is based on preclinical studies showing that blocking μ opioid activation exacerbates distress, intensifies social comfort seeking, and inhibits the relieving effects of social comfort. Conversely, drugs that promote μ opioid activation can alleviate distress even in the absence of social interaction and reduce social approach behavior. Exposure to opioid drugs during distress could therefore hijack a fundamental neurobiological mechanism for social attachment and turn it into a pathway for addiction. **(B)** In a state of homeostatic balance, appetitive motivation promotes social exploration and affiliation. SOMSOM posits that μ opioid signaling also modulates this motivation. This is based on studies showing that reduced μ opioid activation suppresses responses to social reward and reduces social approach, while increased activation promotes social exploration and social reward responsiveness. [Figure is adapted with permission from Løseth *et al*. (88).]

We have previously proposed the state-dependent μ opioid modulation of social motivation (see Figure 3) model to explain the contrasting effects of opioids in distressed animals (88). Human research on the topic is still scarce. Below we explore the question of how chronic opioid use may impact social homeostatic processes and loneliness.

### Loneliness And Stigma In Oud

Loneliness, social isolation, and low satisfaction with the quality of contact with family and friends are prevalent among individuals with OUD (15,136). Patients in medication-assisted treatment for OUD typically report having smaller and weaker social networks than healthy community control individuals (137), perceive themselves as having lower social status and receiving more criticism from others (137), feel isolated from peers and themselves (self-alienation), and experience feelings of inadequacy in social situations (138). Poor self-esteem and self-stigma are likely corroborated by the social challenges faced by individuals with OUD, who are stereotyped as dangerous and unpredictable (139). OUD is highly stigmatized, often viewed as a result of personal failure, and frequently leads to significant social exclusion including from government assistance for basic needs such as food or housing (140). This heightened level of social threat may compound loneliness by reducing social initiative and trust, which in turn increases risk of further opioid use, particularly when drug use occurs in a social context. Social support within a drug-using community can even work to establish and maintain addiction (141,142).

Chronic opioid exposure can disrupt production of endogenous opioids and μ opioid receptor function, resulting in opioid-induced hyperalgesia, analgesic tolerance, and negative emotions (143). How social homeostatic mechanisms may be impacted by these changes remains an open question. In rodents, prolonged exposure to opioid drugs produces behavioral changes that have been interpreted as social indifference (144). Social homeostatic imbalance is a maintaining factor in OUD. The most commonly endorsed reasons for drug relapse are lack of social support and feelings of loneliness (145,146). Patients in methadone treatment report that a desire to self-medicate the social pain of loneliness motivated on-top illicit opioid use (136). This is consistent with brain opioid theory of social attachment’s postulation that opioid drugs offer temporary relief from social distress but highlights how they cannot replace the need for social connection. Instead, opioid drug use may exacerbate loneliness and social deficiency by preventing pursuit of social contact, perhaps leading to a kind of hyperisolation akin to the hyperalgesic and hyperkatifeic effects described by Koob (23). According to the allostatic model of addiction, the subjective experience of pain and negative affect worsens over time, even when acute drug effects reduce the aversive motivation (23). In contrast, boosting the psychosocial resources available to people with OUD promises to improve social homeostatic balance and reduce loneliness-motivated drug use through increased social utility and self-regulation capacity.

### Importance Of Social Connection In Treatment Of Drug Use Disorders

Engaging in social activities that foster social connectedness and provide access to social support promotes drug abstinence (147), as do interventions that enhance feelings of love in general (148). Perceived availability of social support predicts improved recovery trajectories in patients on both drug maintenance (149) and antagonist treatment (150,151), which suggests that the beneficial social effect is not prevented by pharmacological treatment. Successful drug use cessation involves changing the social network and severing ties to active drug users (152,153). Building relationships with abstinent peers can reduce loneliness and instill a sense of connectedness, acceptance, and belonging that is highlighted as an important resource during OUD recovery: “At last I found someone who had walked in my shoes. It was such a relief not to be alone anymore.” and “It was so important that the women here never rejected me.” (154). The success of 12-step programs and mutual support groups for substance use disorders in general underscores the critical role of social bonds in motivation for recovery (155). In Norway, mothers with OUD in stable opioid maintenance treatment with extensive support from health care and social services were able to maintain custody of their children and had normal levels of reward sensitivity (156), and their children had markedly better mental health status than those living in foster care (157). Tailoring treatment to bolster individuals’ social life and reduce loneliness promotes successful, long-lasting recovery. Social prescribing to community groups could be one fruitful avenue to capitalize on these effects (158). However, social interventions alone may be insufficient for treating addiction because its underlying mechanisms extend beyond social homeostasis. Combining interventions that boost social connection with interventions promoting emotion regulation in a social context (159) may be especially efficacious, including for patients in medication-assisted treatment (149).

## Conclusions And Future Directions

The increased risk of OUD in lonely individuals may well be related to opioids’ ability to modulate social homeostatic processes, but these links warrant further empirical exploration. The evidence on opioid regulation of social behaviors in healthy humans is still limited, but emerging patterns fit with a state- and context-sensitive role of μ opioids in maintaining balance between social approach and avoidance. μ Opioids may impact social processes by 1) increasing sensitivity to positive aspects, 2) decreasing perception of threat, or 3) regulating homeostatic set point. Effects of acute opioid manipulations in humans are typically subtle. Whether they could amount to larger impact on social behavior and connectedness over time is unclear but remains a possibility.

Emery and Akil used the metaphor of a spinning plate in their review of the literature: A perturbation in any part of the μ opioid system could bring the whole system out of balance (160). However, the neuroplastic changes caused by chronic exposure prevent generalization from acute drug studies to chronic opioid use. How social homeostasis influences and is impacted by opioid drug use in the short- and long-term would be addressed best in longitudinal studies that capture the progression from initial to chronic use. Ecological momentary assessment is a promising technique to study complex real-life social dynamics. Patients who are about to be exposed to opioids (e.g., waiting for surgery) are an interesting candidate group for longitudinal study due to the risk of postsurgical opioid misuse. Moreover, psychopharmacological studies using ecologically valid tasks could explore how acute doses of opioid agonist or antagonists affect social homeostasis during in-person interactions between friends, romantic partners, and strangers in threatening or rewarding contexts. Furthermore, more direct translation of preclinical findings to humans could help bridge the gap between mechanistic data and clinical research.

## Figures and Tables

**Figure 1:**
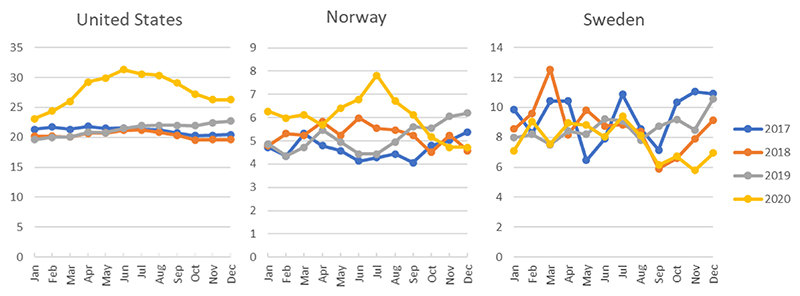
Drug-overdose mortality per 100,000 people by month and year. Rates are annualized. United States and Norway graphs were adapted with permission from Friedman and Gjersing (2). Sweden numbers were retrieved by the authors directly from The Public Health Agency of Sweden (161).

## Box 1 The Question of Generalizability From Nonhuman to Human Findings

As reviewed here, human opioid drug studies do not unequivocally support the standard interpretation of the evidence from model systems, i.e., that endogenous and exogenous opioids promote reward and inhibit pain across domains. Notably, prominent effects of opioid agonists such as increased locomotion in rodents (165) are not observed in humans, who report opioids as sedative [and often aversive (166)]. Conversely, systemic opioid blockade induces conditioned place aversion in rodents (167,168) but no consistent mood effects in healthy humans (169). Only a minority of healthy study participants report euphoria and/or decreased stress from acute opioids (104–106,170,171). Moreover, while opioid medications relieve pain across species, in humans, their analgesic effects are highly variable and unpredictable (172–174).

On the other hand, human neuroimaging studies support the existence of preserved opioid-sensitive microcircuits that were originally described in rodents. Functional magnetic resonance imaging in combination with both opioid agonist and antagonist treatment implicates the descending pain inhibitory system in pain regulation (175,176). In both rodents and humans, opioids are thought to inhibit the antireward circuit centered on the habenula (177,178), whereas an opioid-sensitive affective gradient (from threat to reward) described in the rat ventral striatum (179) also appears to have its parallel in the human brain (180,181). Therefore, we conclude that while the generalizability to drug use and addiction may be limited, there is good reason to believe that the microcircuits for social homeostasis described in mice hold some translational value for understanding social homeostatic processes in the human brain. Moreover, because drugs bind indiscriminately to μ receptors across microcircuits that promote different and sometimes opposing behaviors, the human psychopharmacological approach could in theory underestimate the effects of endogenous opioids acting within discrete microcircuits to promote reward-related behaviors.
